# Comparison of conventional orthodontic method with corticotomy and piezocision during rapid canine retraction

**DOI:** 10.6026/973206300210324

**Published:** 2025-03-31

**Authors:** Ambalika Shrotey, Arvind Nair, Virendra Vadher, Shweta Singh, Vineeta Gupta, Chhaya Barapatre

**Affiliations:** 1 Department of Orthodontics and Dentofacial Orthopedics, Government Dental College, Raipur, Chhattisgarh, India; 2 Department of Periodontics, Government Dental College, Raipur, Chhattisgarh, India

**Keywords:** Orthodontic tooth movement, corticotomy, piezocision, canine retraction, regional acceleratory phenomenon (RAP)

## Abstract

The effect of corticotomy and piezocision to move canines back into position using a split-mouth approach is of interest. Hence, 18 patients were included with their upper first premolars extracted and corticotomy was performed on one side of the upper jaw and piezocision on the other side, with the opposite sides serving as controls. The average movement of the canines was significantly greater in the areas where corticotomy (2.14 ± 0.65 mm) and piezocision (2.20 ± 0.54 mm) was used compared to the control sides (p < 0.05) and pain scores were higher for the corticotomy group at the 24-hour mark (p = 0.001), while there were no significant changes in the inclination of the canines (p>0.05) by the end of the 12 weeks. Piezocision resulted in less post-operative discomfort even through both methods successfully sped up the movement of canines.

## Background:

Orthodontic treatment is widely recognized for its effectiveness in correcting malocclusions and improving oral function. However, one of the most significant challenges in orthodontic therapy is the prolonged treatment duration, which typically extends from 18 to 24 months [1]. Prolonged durations of orthodontic treatment can boost the possibility of complications, which includes root resorption, gum disease, tooth decay and the development of white spots on teeth. In addition, people might encounter a greater challenge in maintaining motivation and dedication to their treatment plan [2, 3]. Traditional orthodontic approaches essentially depend on mechanical forces to promote bone remodelling, that involves bone resorption on the pressure side and bone deposition on the tension side of the tooth. Still, this process is gradual and could be influenced by the density and remodelling ability of alveolar bone [4, 5]. Many methods have been developed for accelerating orthodontic tooth movement using invasive, minimally invasive and non-invasive approaches. Surgeons assisted orthodontics-including methods like corticotomy and piezocision are quite popular approaches [6, 7]. These techniques' ability to maximise tooth movement speed while reducing adverse effects [8] helps to explain their appeal Heinrich Kole presented corticotomy-assisted orthodontics first in 1959 [8]. This approach consists in precisely cutting the cortical bone to enable faster tooth movement. Reducing the resistance from the dense cortical bone helps to increase bone turnover, so enabling teeth to migrate more naturally into their desired placements [9]. Decortication, sometimes known as corticotomy, is the intentional cutting of cortical bone such that the underlying trabecular bone is left whole. This mechanism is thought to start the Regional Acceleratory Phenomenon (RAP), a biological reaction first described by Frost in 1983 that causes temporary localised demineralisation and enhanced bone regeneration following surgical damage [10]. All the while preserving periodontal health, several studies have demonstrated that corticotomy dramatically reduces the length of orthodontic treatment, producing results two to four times faster than conventional techniques [11, 12]. Piezocision and other less invasive procedures are becoming more and more sought for as possible substitutes for conventional corticotomy. Dibart *et al.* (2009) [13] propose piezocision, in which a piezoelectric surgical tool performs cortical micro-incisions in gingival tissues, therefore negating the need for a mucoperiosteal flap. This approach reduces surgical trauma and post-operative problems and promotes regional bone remodelling, hence improving tooth movement [14, 15]. Investigating non-invasive approaches to improve orthodontic tooth movement has been done using pharmacological drugs and devices for physical stimulation [16]. Studies show that thyroxine, vitamin D, parathyroid hormone and prostaglandins may influence orthodontic tooth movement and bone metabolism. Their more general therapeutic use is limited, nonetheless, by their systematic side effects and need for recurrent treatment [15]. Conversely, physical devices such as AcceleDent, Orthopulse and Propel Device have been investigated; nonetheless, their clinical efficacy is still debatable [17]. Therefore, it is of interest to investigate and compare the efficacy of corticotomy and piezocision in accelerating the movement of canines.

## Methods and Materials:

A split-mouth study was conducted in the Department of Orthodontics and Dentofacial Orthopaedics at the Government Dental College in Raipur. Before the start of the study, ethical approval under IEC Proposal No. 4780/GDC/ETHICS COMMITTEE/2022 and received written consent from all participants. The research included 18 volunteers who fulfilled the criteria and were randomly assigned to two groups. Group 1 underwent a corticotomy on one side and a regular orthodontic technique on the other, whereas Group 2 received a piezocision on one side and the standard approach on the opposite side. Patients aged 15 to 25 years who had Class II division 1 malocclusion (with mild or no crowding) or Class I bimaxillary protrusion that required the extraction of the maxillary first premolar were included in the study. All participants had never undergone orthodontic treatment before, maintained good oral hygiene, had probing depth values of 3 mm or less and had enough attached gingiva thickness (between 1-2 mm). Everyone had to agree to participate and provide informed consent. We excluded anyone with skeletal Class III or Class II division 2 malocclusion, severe crowding, or any health issues that could affect bone formation or density, like osteoporosis, vitamin D deficiency, or diabetes. We also ruled out patients with signs of bone loss at the surgical sites, a history of tobacco use in the past year, or long-term use of antibiotics or corticosteroids. All participants received traditional orthodontic treatment, which included leveling and alignment using MBT 0.022" slot brackets and a sequence of Ni-Ti archwires. We conducted radiographic assessments with lateral cephalograms, OPG and Cone-Beam Computed Tomography. The first maxillary premolar on the designated side was extracted a day before the surgery and the other premolar was extracted on the day of the procedure, followed by either corticotomy or piezocision. In the corticotomy group, we made a small buccal incision and created a full-thickness flap, performing vertical cuts and perforations using a piezotome. After that, we repositioned and sutured the flap. For the piezocision group, we made micro-incisions and vertical cortical cuts without elevating the flap, which helped preserve the integrity of the papillae and then sutured it. Immediately after surgery, we placed a 0.019 x 0.025" stainless steel wire and applied a force of 150 g from nickel-titanium closed-coil springs on both sides for canine retraction. A transpalatal arch helped stabilize the molars and we started retraction immediately to take advantage of the Regional Acceleratory Phenomenon (RAP). We followed up biweekly for three months, taking alginate impressions before surgery and at each visit to monitor canine movement. We measured anteroposterior crown tip movements at six different time points (T1-T6) and used Cone-Beam Computed Tomography scans to evaluate changes in canine inclination. Pain levels were recorded using a Visual Analog Scale (VAS) at 24 hours and one week after surgery.

## Statistical analysis:

SPSS software was used to carry out the statistical analysis, setting significance threshold at a p-value of less than 0.05. We examined the data from the measurements and pain assessment scores with the right statistical tests to see if there were any meaningful differences between the groups.

## Results:

The study involved 18 patients aged between 15 and 25 years, with nine individuals randomly placed in each group. We employed a split-mouth study design, where we randomly assigned corticotomy to one side of the upper jaw and piezocision to the other side. The opposite sides acted as controls for both groups.

## Rate of canine retraction:

In the corticotomy group, we found that the experimental side showed a noticeably higher average movement of the canine compared to the control side at different time points. By the 12th week (T6), the experimental side had an average movement of 2.14 ± 0.65 mm, while the control side measured 1.48 ± 0.35 mm, which was statistically significant (p = 0.018). Similarly, in the piezocision group, the experimental side also demonstrated more movement than the control side. At T6, the experimental side recorded 2.20 ± 0.54 mm, compared to 1.48 ± 0.52 mm for the control side (p = 0.012), as shown in Table 1. When comparing the mean rates of canine retraction at experimental sites between the corticotomy and piezocision groups, the corticotomy group initially exhibited a higher rate from T1 to T3 shown in Figure 1 (see PDF). However, from T4 onwards, the piezocision group demonstrated a greater mean retraction rate. Overall, this difference was not statistically significant.

## Pain perception (VAS scores):

Figure 2 (see PDF) depicts pain perception 24 hours (P1) and 1 week (P2) post-procedure. The corticotomy group experienced significantly higher pain (P1: 3.44 ± 0.52 mm) compared to piezocision (P1: 2.44 ± 0.72 mm) (p = 0.001). At P2, pain levels decreased but remained significantly different between the groups.

## Canine inclination:

No statistically significant differences were observed in inclination changes between experimental and control sides within or between groups (p > 0.05).

## Discussion:

The present study aimed to evaluate and compare the efficacy of corticotomy and piezocision in accelerating orthodontic canine retraction, as well as to assess associated pain perception and changes in canine inclination.

## Rate of canine retraction:

Our research shows that both corticotomy and piezocision really boost the speed of canine retraction compared to the control sites. By the 12th week (T6), the corticotomy group had an average movement of 2.14 ± 0.65 mm at the experimental site, while the control site only saw 1.48 ± 0.35 mm (p = 0.018). The piezocision group was similar, with 2.20 ± 0.54 mm of movement at the experimental site compared to 1.48 ± 0.52 mm at the control site (p = 0.012). These findings support research by Viwattanatipa *et al.* [18] which found that corticotomy can speed up tooth movement by 2 to 4 times and piezocision can double the rate compared to traditional methods. Upon comparison, the corticotomy group had a more rapid retraction rate from T1 to T3. Beginning at T4, the piezocision group showed a better average retraction rate. This difference was not statistically significant. This corresponds with a systematic review by Han *et al.* [19], which reported that both corticotomy and piezocision efficiently accelerate orthodontic tooth movement, with no significant differences in efficacy. Abbas *et al.* [20] also found that corticotomies facilitated quicker canine movement compared to piezocision at four distinct time intervals. A thorough evaluation by Lipani *et al.* [21] demonstrated that corticotomy accelerated canine retraction by 1.5 to 4 times, whereas piezocision resulted in retraction that was 1.5 to 2 times faster. Both methods were instrumental in facilitating upper canine retraction in extraction cases.

## Pain perception:

Pain perception using the Visual Analog Scale (VAS) was measured at two points: 24 hours (P1) and 1 week (P2) after the procedure. The corticotomy group reported noticeably more pain at P1, scoring an average of 3.44 ± 0.52 mm, while the piezocision group had a lower average of 2.44 ± 0.72 mm (p = 0.001). The difference between the two groups was still significant even though pain levels dropped by P2. These results align with previous research by Viwattanatipa *et al.* [18] which suggests that piezocision leads to higher patient satisfaction and potentially less discomfort after surgery compared to corticotomy.

## Canine inclination:

In our study both the corticotomy and piezocision techniques do not negatively impact the axial inclination of the canines during retraction. This finding matches up with the study by Alfawal *et al.* [22], which reported that neither technique causes notable changes in tooth inclination.

## Limitations and future research:

The study has some limitations, such as a small sample size and a short follow-up period. To really confirm these findings and see how corticotomy and piezocision affect periodontal health and treatment outcomes in the long run, we need future research with larger groups and longer observation times.

## Conclusion:

Both corticotomy and piezocision are great options for speeding up the movement of canines in orthodontics without negatively impacting how the teeth are angled. However, piezocision tends to cause less pain; it might be the better choice for patients who value comfort. Clinicians should consider the advantages of quicker tooth movement alongside how comfortable the patient feels and how invasive the procedures are when deciding which method to use.

## Declarations:

## Author's contribution:

All authors contributed significantly to the study. Ambalika Shrotey conceptualized the research, designed the methodology and led data collection. Arvind Nair supervised the study, validated the methodology and performed statistical analysis. Shweta Singh conducted the literature review, interpreted data and contributed to manuscript writing. Vineeta Gupta was responsible for clinical data collection, patient management and surgical procedures. Chhaya handled data analysis, prepared figures and tables and ensured adherence to ethical guidelines. Virendra Vadher conducted the final review, edited the manuscript and facilitated funding acquisition. All authors reviewed and approved the final version of the manuscript.

## Institutional review board statement:

Ethical approval was obtained before initiating the study with IEC Proposal No. 4780/GDC/ETHICS COMMITTEE/2022.

## Informed consent statement:

Informed written consent was acquired from all participants.

## Figures and Tables

**Table 1:** Comparative descriptive statistics for rates of canine retraction at experimental and control sites

**Period (Weeks)**	**Corticotomy (Experimental) (Mean ± SD mm)**	**Corticotomy (Control) (Mean ± SD mm)**	**Piezocision (Experimental) (Mean ± SD mm)**	**Piezocision (Control) (Mean ± SD mm)**	**P-Value (Corticotomy)**	**P-Value (Piezocision)**
2 wk (T1)	0.5111 ± 0.2204	0.3000 ± 0.2000	0.3667 ± 0.3354	0.2222 ± 0.3192	0.049*	0.363
4 wk (T2)	0.8889 ± 0.2713	0.5778 ± 0.2279	0.7333 ± 0.3500	0.5000 ± 0.4062	0.018*	0.214
6 wk (T3)	1.2333 ± 0.3427	0.8333 ± 0.2345	1.0667 ± 0.4387	0.7444 ± 0.3972	0.011*	0.122
8 wk (T4)	1.4556 ± 0.4693	1.0889 ± 0.2758	1.5222 ± 0.3865	1.0222 ± 0.4465	0.06	0.022*
10 wk (T5)	1.7111 ± 0.6372	1.2444 ± 0.3045	1.8444 ± 0.4216	1.2667 ± 0.4743	0.065	0.015*
12 wk (T6)	2.1444 ± 0.6578	1.4889 ± 0.3515	2.2000 ± 0.5431	1.4889 ± 0.5254	0.018*	0.012*
*Statistically significant values (P < 0.05).
